# Development and validation of a nomogram for predicting in-hospital mortality in older adult hip fracture patients with atrial fibrillation: a retrospective study

**DOI:** 10.3389/fmed.2025.1605437

**Published:** 2025-07-23

**Authors:** Zhenli Li, Jing He, Tiezhu Yao, Guang Liu, Jing Liu, Ling Guo, Mengjia Li, Zhengkun Guan, Ruolian Gao, Jingtao Ma

**Affiliations:** ^1^Department of Cardiology, The Fourth Hospital of Hebei Medical University, Shijiazhuang, China; ^2^Department of Cardiology, Anzhen Hospital Affiliated to Capital Medical University, Beijing, China; ^3^School of Basic Medicine, Hebei Medical University, Shijiazhuang, China

**Keywords:** intensive care unit, hip fractures, atrial fibrillation, mortality, nomogram, machine learning

## Abstract

**Background:**

Hip fracture is prevalent among older adult patients, which often results in intensive care unit (ICU) admission. When complicated with atrial fibrillation (AF), older adult patients with hip fractures were observed to have a high short-term mortality. However, few studies have focused specifically on such a cohort. This study aimed to develop and validate a nomogram to evaluate the in-hospital mortality risk of such a group in the ICU.

**Methods:**

We enrolled older adult patients with hip fractures complicated by AF in the Medical Information Mart for Intensive Care Database (MIMIC). Logistic regression (LR) and Least Absolute Shrinkage and Selection Operator (LASSO) algorithms were employed to screen features. We further used Extreme Gradient Boosting (XGBoost) based on features selected by LR and LASSO algorithms to assist in identifying the final model-established features. An Electronic Intensive Care Unit Collaborative Research Database (eICU-CRD) was utilized for external validation. The area under curves (AUC), calibration curves, Delong test, decision curve analysis (DCA), net reclassification improvement (NRI), and integrated discrimination improvement (IDI) were performed to evaluate the prediction performance. Ultimately, a visualized nomogram was constructed to provide convenient access for clinicians to evaluate mortality risk.

**Results:**

A total of 308 patients were enrolled in this study. We employed LR and LASSO algorithms to initially screen out 15 and 20 features, respectively. Next, 10 features, which were the intersection of features selected by the above methods, were further utilized to develop an XGBoost model to obtain the rank of feature importance. Finally, eight features were ultimately selected to develop a nomogram by comparing the AUCs of LR models originating from a “feature-adding by the feature rank” strategy. The nomogram exhibited superior predictive performance (AUC:0.834) than conventional scoring systems in the training set, with an AUC of 0.715 in external validation.

**Conclusion:**

Our study constructed a predictive model based on features selected by machine learning approaches to evaluate the in-hospital mortality risk of critically ill patients with hip fractures combined with AF. An accessible nomogram was offered to facilitate clinical decision-making.

## Introduction

The rapid aging of the population has raised significant concerns about the quality of life for older adults, particularly in relation to their medical care (1). Hip fracture is typically observed in older adult patients, with a global prevalence of 681.35 per 100,000 population in patients over 55 years, which is associated with 5–8 fold increased chance of death during the first 3 months (2, 3). Approximately one-third of patients suffering from hip fractures succumb within the first postoperative year (4). Several studies have highlighted a high mortality risk among hip fracture patients and regarded those over 60 years old as a definition of older adults (5–7).

Due to a series of complications and advanced age, older people with hip fractures are prone to intensive care unit (ICU) admissions. Atrial fibrillation (AF) is the most prevalent arrhythmia among older adults, affecting 5% of those above 65 years. The prevalence of AF increases over the lifetime, peaking at 10% in older adults aged over 80 years (8). With population aging, this prevalence is expected to increase from 5.2 million in 2010 to 12.1 million in 2030 in America (9, 10). Remarkably, accumulating evidence has indicated that AF significantly contributes to adverse clinical outcomes, such as stroke, cognitive impairment, myocardial infarction (MI), and heart failure, and correlates with a 1.5 to 2.0 fold rise in risk of mortality (11). In addition, as related to aging, AF represents a frequent comorbidity for patients in an ICU. Furthermore, an international cohort study has revealed that AF is a predictor for the detrimental prognosis of senile patients in an ICU, associated with more ischemic, thromboembolic, severe bleeding events, and higher mortality (12). Previous studies suggested that AF is associated with hip fracture by raising the incidence of falls, while hip fracture correlates with a 0.4-fold increased risk of AF (13). Meanwhile, AF is also deemed an independent risk factor for mortality in patients with sustained hip fractures. The prevalence of AF in hip fracture patients was reported as 12–15% (14). Therefore, older adult patients sustaining both AF and hip fractures deserve more clinical attention. Nevertheless, there has been a paucity of studies focusing specifically on this cohort. This highlights an urgent clinical need for the development of predictive models for short-term mortality in this population.

Moreover, AF is a common arrhythmia during the perioperative period, which is related to poor outcomes. Adunsky et al. demonstrated that 1-year mortality in older adult patients undergoing hip fracture repair is significantly increased in patients with postoperative AF (15). Leibowitz et al. also demonstrated a high correlation between perioperative AF and 1-year mortality in older adult hip fracture patients. In-hospital mortality of older adult hip fracture patients has been explored in several studies, which is a critical induce reflecting the treatment effect (16, 17); for example, a recent study demonstrated the risk factors of in-hospital mortality for hip fracture patients with AF nationwide, mainly involving sepsis, respiratory failure, liver disorders, and acute kidney injury (5). In addition, the effort to develop prognostic models for older adult hip fracture groups has also been made. For example, Fu et al. developed a nomogram-based model to predict the preoperative AF among older adult patients with HF, and Lu et al. also constructed a nomogram-based model to predict the short-term mortality in older adult hip fracture patients with complicated heart failure in an ICU (18, 19). Nevertheless, few studies have identified the risk factors of in-hospital all-cause mortality for older adult patients with hip fractures complicated by AF from an ICU cohort and developed a dynamic predictive and visual tool for clinical use.

To bridge the clinical gap and enhance physicians’ understanding of risk factors associated with this population, further investigations are warranted. This study attempted to construct a user-friendly prediction model in the form of a nomogram to provide insights into individual risk evaluation and assist in developing tailored therapeutic strategies. As a consequence, we employed a nomogram approach to develop a prediction model for in-hospital mortality probability in ICU patients with hip fractures complicated by AF, using the free and open critical care databases—Medical Information Mart for Intensive Care (MIMIC) database and an Electronic Intensive Care Unit Collaborative Research Database (eICU-CRD).

## Materials and methods

### Data source

We extracted derivation data from MIMIC databases (III and IV), which are known as an open-sourced database with medical health records for patients who have been admitted to Beth Israel Deaconess Medical Center (20, 21). We also extracted data from eICU-CRD, which is composed of 139,367 patients admitted between 2014 and 2015, as the dataset for external validation (22). Structured Query Language (SQL) and pgAdmin4 PostgreSQL 9.6 were used to search for the required data. Moreover, before processing our study, we had completed an online course offered by the National Institutes of Health (NIH), which granted us access to the MIMIC and eICU-CRD database (certification number: 64322113). All methods were carried out in accordance with the “Declaration of Helsinki.” This retrospective study did not use personal identifying information and thus did not require informed patient consent or Institutional Ethics Committee Board approval.

### Study population

**Figure 1:**
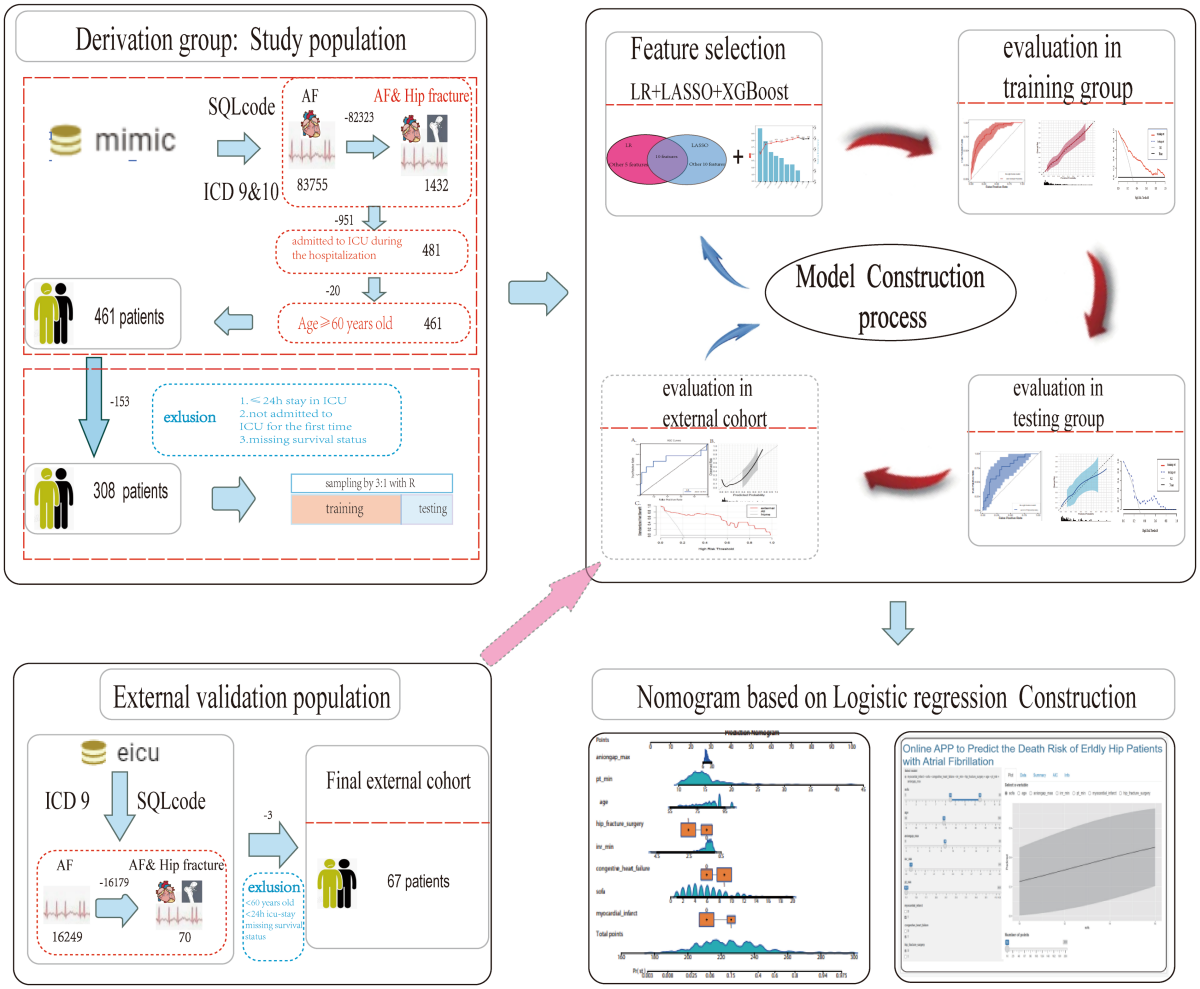
Flow chart of the study design. MIMIC, Medical Information Mart for Intensive Care; eICU-CRD, eICU Collaborative Research Database; ICD, international classification of diseases; HF, hip fracture; AF, atrial fibrillation; ICU, intensive care unit; LASSO, least absolute shrinkage and selection operator; XGboost, extreme gradient boosting; LR, logistic regression; AUC, area under curve.

As shown in Figure 1, patients from the MIMIC database and eICU-CRD were fully traversed. Initially, we included target patients who met the following criteria: (1) diagnosed with hip fracture and AF in MIMIC database by the international classification of diseases (ICD)-9 or -10 version diagnostic code (Supplementary Table S1), (2) age *≥* 60 years old, and (3) admitted to ICU during the hospitalization. Further, patients who had a stay of *≤*24 h in an ICU or were not admitted to an ICU for the first time were excluded. In addition, patients selected from the eICU-CRD were regarded as an independent validation set to evaluate the generalizability of the established models, which were also identified by ICD-9 (Figure 1).

### Predictor variables

Variables extracted from the MIMIC database included demographic characteristics, vital signs, laboratory tests, and co-existing diseases. The “hadmi_id” parameter was used to extract demographic characteristics from the MIMIC database, while “patientunitstayid” was used for those from the eICU-CRD database, including age, sex, weight, and ethnicity. In terms of comorbidities, chronic pulmonary diseases (COPD), renal disease, severe liver disease, peripheral vascular disease (PVD), myocardial infarction (MI), acute heart failure, cerebrovascular disease, dementia, and cancer-related comorbidities were mainly extracted. The vital sign values, including heart rate (HR), systolic blood pressure (SBP), respiratory rate (RR), saturation of peripheral oxygen (Spo2), temperature, and urine output (UO), were extracted (e.g., MIMIC: “first_day_vitalsign” chart) and presented in the suitable format accordingly. The laboratory results included anion gap, bicarbonate, creatinine, chloride, glucose, blood urea nitrogen, potassium, partial thromboplastin time (PTT), prothrombin time (PT), International normalized ratio (INR), hematocrit, hemoglobin, white blood cell (WBC) count, and platelets. Furthermore, Sequential Organ Failure Assessment (SOFA), Glasgow Coma Scale (GCS), and Acute Physiology Score (APS) III scores were extracted, while the CHA2DS2-VASc score and HAS-BLED score were calculated for each patient. Ventilation, vasopressin, and anticoagulant drug treatments were also included on the first day. The primary outcome of our study was all-cause mortality during hospitalization. Laboratory tests and vital signs were measured within the first 24 h after ICU admission. Variables with a missing value proportion of more than 20% were excluded, such as albumin, bilirubin, and D_dimer. The exact missing proportion of each variable can be found in Supplementary Table S2. Categorical variables and continuous variables were presented in a suitable data format accordingly.

### Imputation of missing values

After extracting variables with missing value proportion less than 20%, we used the KNNImputer (KNN) method with a “n_neighbors = 5” parameter to impute the original data, which has advantages of simplicity, non-parametric nature, preservation of data structure, adaptability to numerical and categorical data, robustness to outliers, no need for model training, and customizable parameters. The above method was based on the assumption that all missing values are missing at random (MAR). This decision aligns with the suitability of KNNImputer for MAR scenarios.

### Statistical analysis

Baseline data and clinical outcomes in the training and validation cohorts were expressed. Categorical variables were expressed as percentages and compared by the chi-square test or Fisher’s exact test accordingly. Continuous variables were presented as the mean with standard deviation (SD) or the median with interquartile range (IQR), according to whether a variable had a normal distribution after Shapiro–Wilk tests were used. T-test and Wilcoxon Rank-Sum Test were used to compare continuous variables. Data cleaning and transformation, variable selection, model building, performance evaluation, and validation were all conducted in R software (version 4.4.3) using appropriate R packages (e.g., “fastshap”). Logistic regression (LR) and Least Absolute Shrinkage and Selection Operator (LASSO) algorithms were employed to screen features. Extreme Gradient Boosting (XGBoost) algorithm was used to screen the most important features. The area under the curves [AUC (which is equal to concordance index in this study)], calibration curves, Delong test, decision curve analysis (DCA), net reclassification improvement (NRI), and integrated discrimination improvement (IDI) were performed to evaluate the prediction performance. All tests were two-sided, and *p ≤* 0.05 was considered statistically significant.

## Results

### Baseline characteristics

A total of 308 patients from the MIMIC database were included for model derivation in this study. The patients were divided into training and internal validation groups using completely randomized sampling, with a ratio of 3:1. No statistically significant differences were found between the training cohort and internal validation (testing) cohort in most terms. The baseline characteristics of the patients are presented in Table 1. The baseline characteristics of these patients, comparing the survival and non-survival groups, are presented in Supplementary Table S3, where a significant difference is observed. In addition, 67 patients from eICU-CRD were extracted as the external validation cohort, with nine dead cases and 58 survivors. The baseline characteristics of the patients between the MIMIC and eICU-CRD cohorts are shown in Supplementary Table S4. The usage of anticoagulant and antiplatelet drugs among all patients in the training set is depicted in Supplementary Figure S1A. We also compared the usage of anticoagulant and antiplatelet drugs between the MI and non-MI groups in the training set (Supplementary Figure S1B).

**Table 1:** Characteristics of the patients divided into training and validation cohorts.

Characteristic	Training cohort	Validation cohort	*p* value
	*N* = 231	*N* = 77	
Basic information			
Age (Median, IQR)	83 (75, 88)	81 (74, 86)	0.216^a^
Gender			0.231^c^
Female	102 (44.2%)	28 (36.4%)	
Male	129 (55.8%)	49 (63.6%)	
Race			0.936^c^
Others	49 (21.2%)	16 (20.8%)	
White	182 (78.8%)	61 (79.2%)	
Weight (Median, IQR)	67 (60, 80)	68 (56, 82)	0.645^a^
Clinical score system			
SOFA (Median, IQR)	5.0 (3.0, 8.0)	5.0 (3.0, 7.0)	0.453^a^
GCS (Median, IQR)	14.00 (13.00, 15.00)	14.00 (13.00, 15.00)	0.685^a^
APS III (Median, IQR)	48 (38, 61)	48 (42, 57)	0.921^a^
Comorbidities			
Myocardial infarct			0.403^c^
No	176 (76.2%)	55 (71.4%)	
Yes	55 (23.8%)	22 (28.6%)	
Peripheral vascular disease			0.042^c^
No	195 (84.4%)	72 (93.5%)	
Yes	36 (15.6%)	5 (6.5%)	
COPD			0.891^c^
No	149 (64.5%)	49 (63.6%)	
Yes	82 (35.5%)	28 (36.4%)	
Mild liver disease			>0.999^b^
No	222 (96.1%)	74 (96.1%)	
Yes	9 (3.9%)	3 (3.9%)	
Renal disease			0.145^c^
No	160 (69.3%)	60 (77.9%)	
Yes	71 (30.7%)	17 (22.1%)	
Rheumatic disease			>0.999^b^
No	220 (95.2%)	74 (96.1%)	
Yes	11 (4.8%)	3 (3.9%)	
Cerebrovascular disease			0.234^c^
No	199 (86.1%)	62 (80.5%)	
Yes	32 (13.9%)	15 (19.5%)	
Congestive heart failure			0.423^c^
No	93 (40.3%)	35 (45.5%)	
Yes	138 (59.7%)	42 (54.5%)	
Dementia			0.322^c^
No	197 (85.3%)	62 (80.5%)	
Yes	34 (14.7%)	15 (19.5%)	
Diabetes with complications			0.441^c^
No	206 (89.2%)	71 (92.2%)	
Yes	25 (10.8%)	6 (7.8%)	
Treatment			
Warfarin			0.508^c^
No	165 (71.4%)	58 (75.3%)	
Yes	66 (28.6%)	19 (24.7%)	
Antiplatelet therapy			0.689^c^
No	135 (58.4%)	43 (55.8%)	
Yes	96 (41.6%)	34 (44.2%)	
Heparin			0.328^c^
No	73 (31.6%)	29 (37.7%)	
Yes	158 (68.4%)	48 (62.3%)	
NOAC			0.314^c^
No	193 (83.5%)	68 (88.3%)	
Yes	38 (16.5%)	9 (11.7%)	
Ventilation_first_day			0.441^c^
No	58 (25.1%)	16 (20.8%)	
Yes	173 (74.9%)	61 (79.2%)	
Vasopressor_first_day			0.602^b^
No	228 (98.7%)	75 (97.4%)	
Yes	3 (1.3%)	2 (2.6%)	
Prognostic information			
In-hospital death			0.690^c^
No	182 (78.8%)	59 (76.6%)	
Yes	49 (21.2%)	18 (23.4%)	

a Wilcoxon rank sum test.

b Fisher’s exact test.

c Pearson’s Chi-squared test.

Continuous variables are expressed in terms of the median with interquartile range. Categorical variables are presented in terms of %.

### The process of nomogram construction

**Figure 2:**
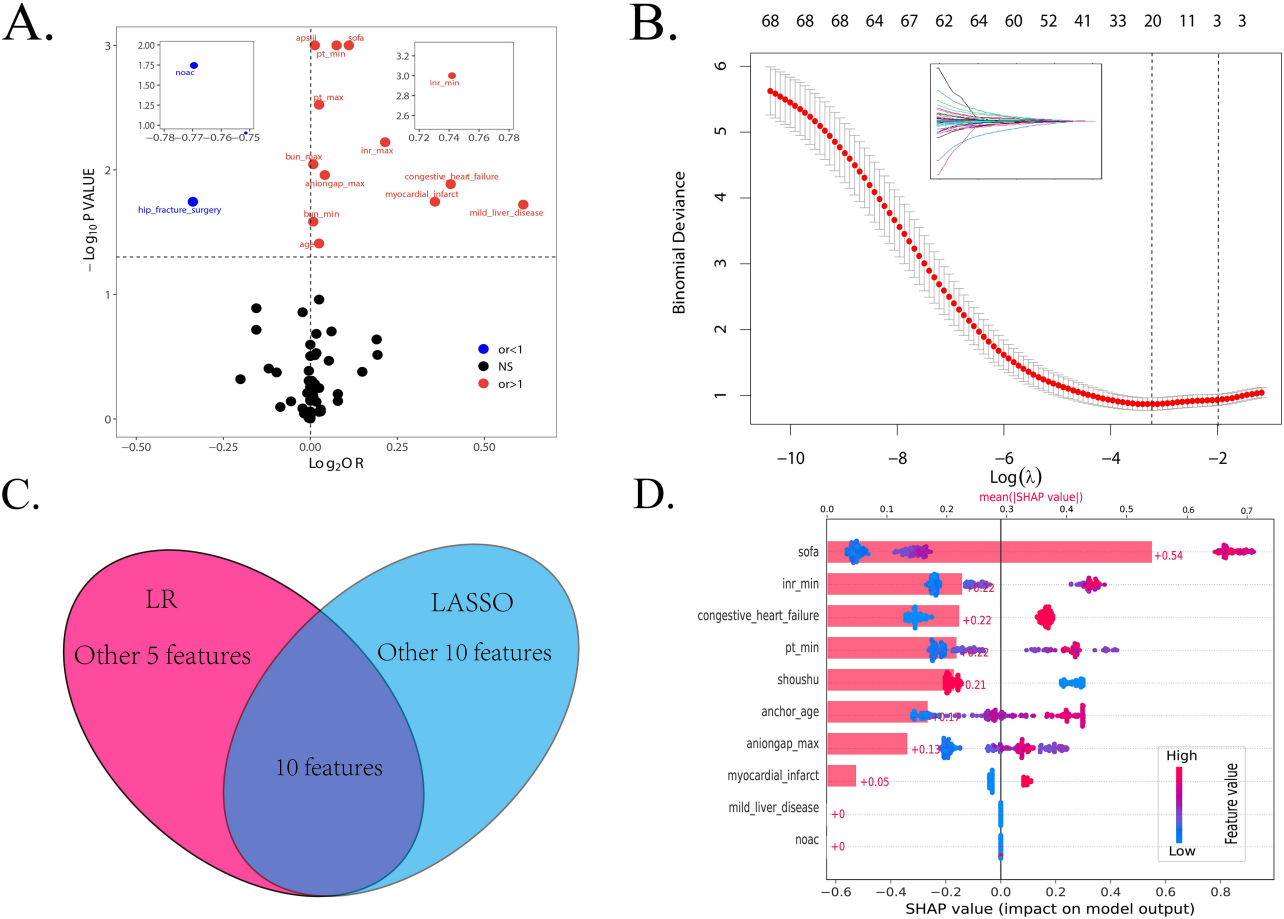
The procession of initially important features screening. **(A)** The result of logistic regression to find the features with a < 0.05 *p* value between all variables and in-hospital mortality. **(B)** The result of LASSO regression to shrink features. **(C)** The intersection of the features selected by the LR and LASSO methods. **(D)** The SHAP analysis based on the XGBoost algorithm. LR, logistic regression; LASSO, least absolute shrinkage and selection operator; OR, odds ratio.

**Figure 3:**
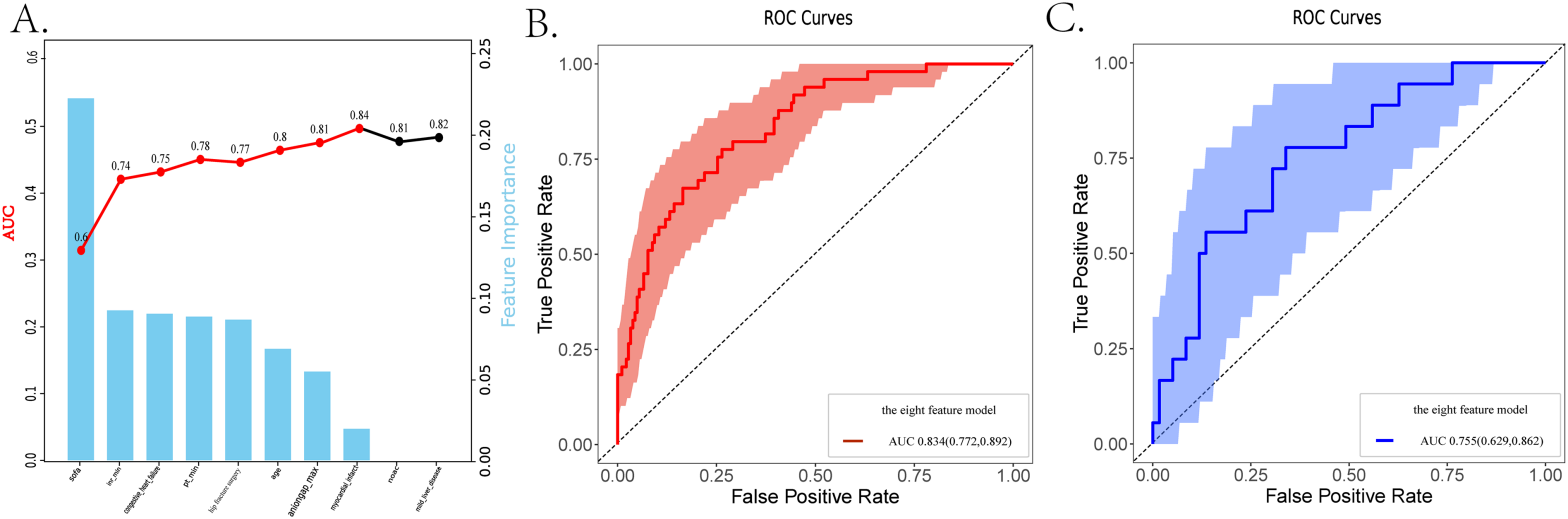
The dynamic feature selection process and the receiver operating characteristic (ROC) analysis are used to evaluate the performance of different models. **(A)** The feature importance based on the XGboost algorithm and the AUCs of different LR models, ranked by the ten selected features. **(B,C)** The ROC analysis with 1,000 repetitions of the nomogram based on the top 8 features in both the training and internal validation cohorts. LR, logistic regression; LASSO, least absolute shrinkage and selection operator; XGboost, extreme gradient boosting; ROC, receiver operating characteristic; AUC, area under the curve.

**Figure 4:**
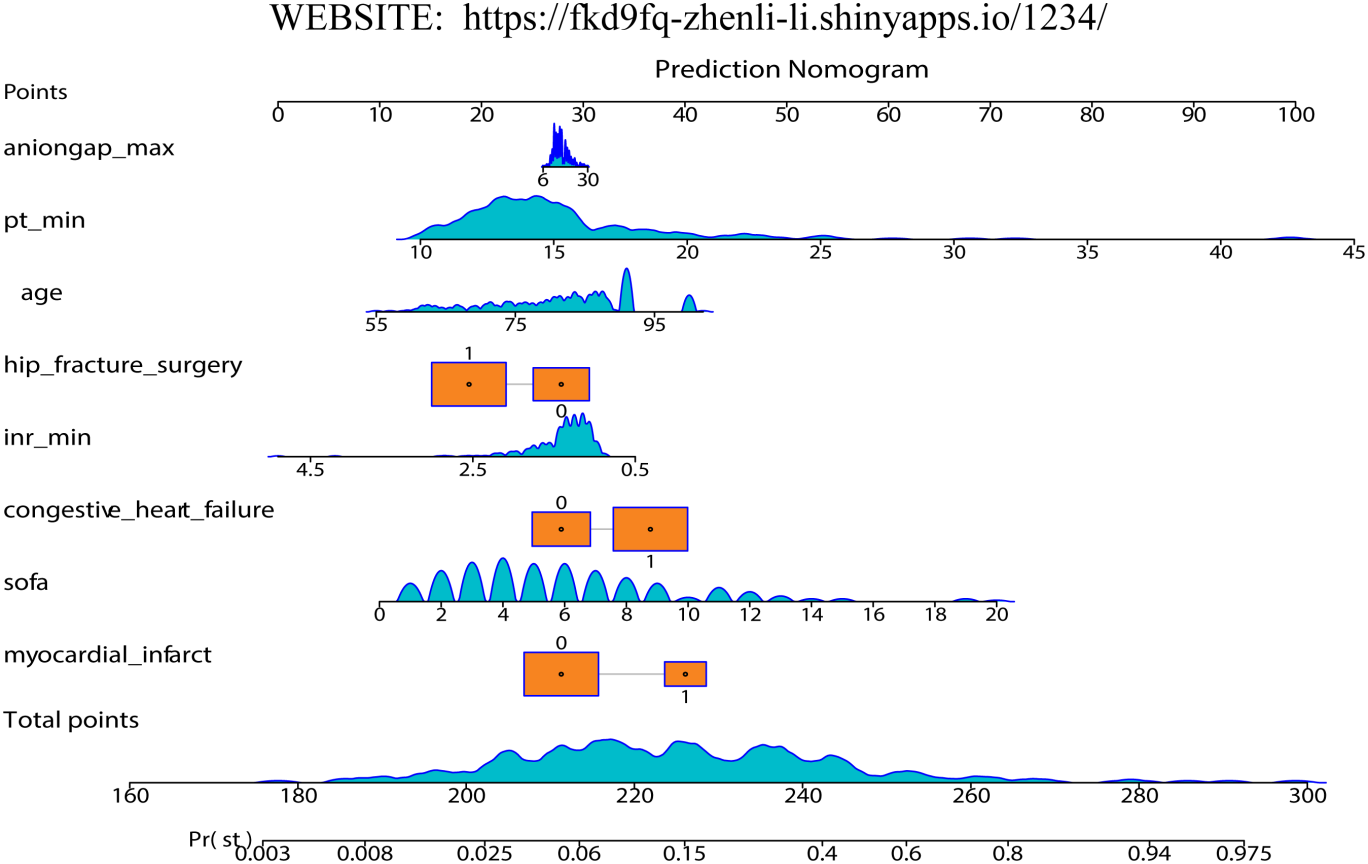
The established nomogram to predict mortality risk for clinical use.

Firstly, we randomly divided patients into the training cohort for model construction and another validation cohort for model validation by a ratio of 3:1. Subsequently, utilizing the training data, we processed the LR analysis and LASSO regression analysis to initially select the most relevant indicators, respectively. Fifteen features with a *p* value <0.05 were screened using the univariate logistic regression (Figure 2A). During the LASSO regression analysis, 20 features were identified by a Lambda.1se strategy with a tenfold cross verification (Figure 2B). In order to evaluate the importance and contribution of variables from the intersection of the 10 features selected by LR and LASSO analysis, we constructed a XGboost machine learning model using the default hyperparameters (Figures 2C,D). As shown in Figure 2D, SHAP analysis was also performed. The ten features ranked as follows: SOFA score, INR_min, congestive heart failure, PT_min, hip fracture surgery, age, anion gap_max, myocardial infarct, mild liver disease, and NOAC therapy (Figure 3A). Afterward, we employed a series of multiple multivariable LR models, which were established by consecutively adding additional one more predictor to the previous model at each time (from 1 feature to 10 features). The final number of involved features was determined by achieving the best performance in the area under the receiver operating characteristic curve (AUC), resulting from 5-fold cross-validation. As shown in Figure 3A, we arbitrarily chose the top eight predictors for further model development (AUC_mean = 0.84), as no incremental performance was observed after adding more features. With the top 8 features, we further constructed a clinical prediction model using multivariable logistic analysis using the training set and visualized it in the format of a nomogram (23) to evaluate the short-term mortality risk of such a group in an ICU (Figure 4).

### Nomogram evaluation

Firstly, we evaluated the predictive performance of the nomogram using ROC analysis of bootstrap with 1,000 repetitions in both training and testing cohorts. It owned a favorable AUC of 0.834 (95% CI:0.772–0.892) in the training set and an acceptable AUC of 0.755 (95% CI:0.629–0.862) in the testing set (Supplementary Figures S3B, C). An AUC of 0.715 was also observed in the eICU-CRD cohort for the external validation (Supplementary Figure S3A). As shown in Supplementary Figure S1, Figure 3B, and Table 2, the metrics called AUCs, Delong tests, NRI, and IDI were further utilized to compare the nomogram’s performance with conventional scoring systems, including APS III, SOFA scores, and HAS-BLED, CHA2DS2-VASc scores. In the training cohort, the AUCs of the above existing evaluation systems for the in-hospital mortality of older adult patients with both hip fractures and AF are, respectively, 0.659, 0.723 and 0.565, 0.583 (Supplementary Figure S2). As indicated in Table 2, statistically significant differences (*p* < 0.05) were observed when the DeLong test was conducted to confirm the nomogram’s superiority compared with the above score systems among the training set. Moreover, in the categorical and continuous NRI analysis for the nomogram compared to other score systems, significant improvements were still displayed in the discriminative performance to correctly classify patients into risk categories [except SOFA vs. nomogram by NRI (categorical)]. Similarly, IDI values further confirmed the superior improvement of the nomogram to differentiate between survival and non-survival groups in this critically ill group.

**Table 2:** Comparison of the nomogram with SOFA, APS III, CHA2DS2-VASc, and HAS-BLED scoring systems in predictive efficiency in the training set.

Comparative method	Estimate / Z or D for DeLong test	95% CI	*p*-value
DeLong test			
Nomogram vs. CHA2DS2-VASc	4.9656	–	<0.001
Nomogram vs. APS III	3.1468	–	0.002
Nomogram vs. SOFA	2.1275	–	0.034
Nomogram vs. HAS-BLED	3.1468	–	0.002
NRI (Categorical)			
Nomogram vs. CHA2DS2-VASc	0.2881	0.1538–0.4223	<0.001
Nomogram vs. APS III	0.2881	0.1538–0.4223	<0.001
Nomogram vs. SOFA	0.0502	−0.0831 to 0.1835	0.460
Nomogram vs. HAS-BLED	0.2881	0.1538–0.4223	<0.001
NRI (Continuous)			
Nomogram vs. CHA2DS2-VASc	0.9749	0.6977–1.2521	<0.001
Nomogram vs. APS III	1.0157	0.7442–1.2872	<0.001
Nomogram vs. SOFA	0.81	0.5276–1.0925	<0.001
Nomogram vs. HAS-BLED	1.0157	0.7442–1.2872	<0.001
IDI			
Nomogram vs. CHA2DS2-VASc	0.265	0.1848–0.3451	<0.001
Nomogram vs. APS III	0.2726	0.1912–0.3539	<0.001
Nomogram vs. SOFA	0.1284	0.068–0.1888	<0.001
Nomogram vs. HAS-BLED	0.2726	0.1912–0.3539	<0.001

CI, confidential interval; NRI, net reclassification improvement; IDI, integrated discrimination improvement.

**Figure 5:**
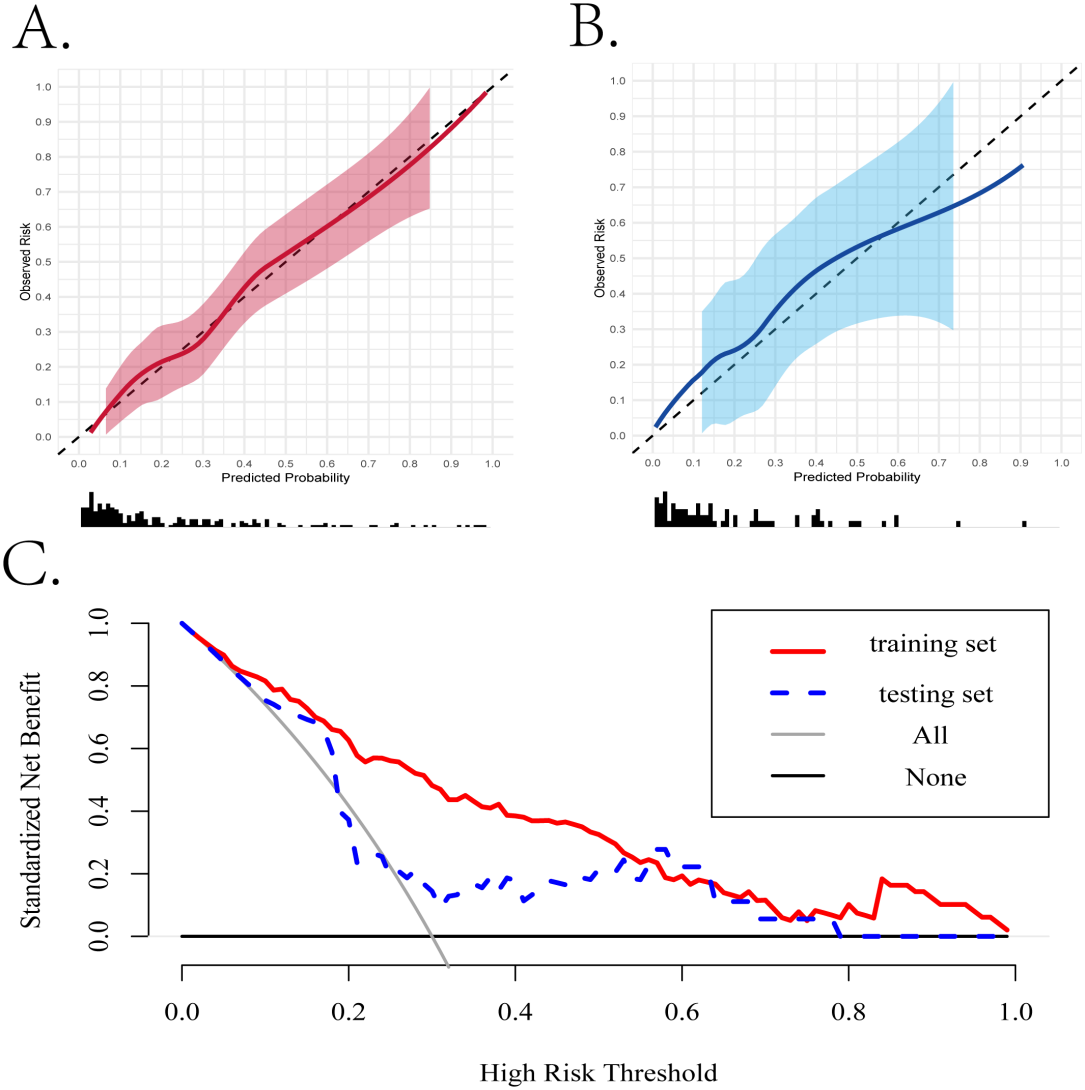
The evaluation of the performance of the nomogram based on the simplified model. **(A,B)** The calibration curves of the training and internal validation cohorts. **(C)** The clinical decision curves in the training and internal validation cohorts.

Subsequently, we assessed the performance of the nomogram model using calibration curves. The curves closely fit the 45-degree diagonal line, whether in the training set and internal validation set, demonstrating a relatively high accuracy and reliability of the model’s predictive ability (Figure 5). Moreover, there is still a relatively good matching degree in almost every interval of predictive probability (Supplementary Figure S3B).

### Clinical application

When assessing the nomogram from a clinical perspective, we used DCA curves to evaluate the clinical benefits of the nomogram in the training set and internal validation set. As shown in Figure 5C, the nomogram model obtains clinical benefit within the threshold probabilities ranging of 5–100% in the training set and 10–80% (except 20–25%) in the internal validation set. When it comes to the external validation set, a risk threshold (5–100%) for obtaining clinical benefit was also observed (Supplementary Figure S3C). Finally, in order to facilitate the clinical application and promotion of the construct nomogram, we have built a web app based on the constructed nomogram simultaneously on https://fkd9fq-zhenli-li.shinyapps.io/1234/, which can output prediction probabilities of all-cause in-hospital death after ICU admissions (Supplementary Figure S4). In the user-friendly interface, clinicians can assess mortality risk using baseline information from critically ill patients and incorporate additional covariates to evaluate how mortality risk varies according to clinical demands.

## Discussion

### The feasibility of the present study

AF is often deemed as a marker of disease severity rather than a direct contributor to mortality (24). Zhang et al. demonstrated that new-onset AF was associated with an increased risk of mortality among ICU patients (25). As common geriatric comorbidities, hip fracture and AF represent significant risk factors associated with mortality (2, 8). To our knowledge, although the relationship between hip fractures and incident AF remains controversial, it displays a higher morbidity in older patients with hip fractures. Moreover, hip fracture patients complicated by AF are more likely to encounter terrible clinical outcomes. Consequently, early evaluation of prognostic hazard for this population, especially in an ICU, is beneficial in guiding clinical practice. Nevertheless, existing prediction models and scoring systems primarily focus on patients with hip fractures or AF, respectively. There remains an urgent need for a risk predictive model tailored for the hip fracture population with concomitant AF.

In this study, based on MIMIC databases, LR and LASSO algorithms were implemented to select significant predictors. Moreover, machine learning, a data-driven tool, offers significant advantages in constructing predictive models due to its excellent ability to handle complex, high-dimensional data and identify intricate patterns that traditional statistical approaches may overlook (26, 27). Thus, we employed the XGBoost algorithm to assist in ranking the top 10 important features and used the SHAP method to overcome the “black box” attribute of the ML method. Finally, a visualized multivariable LR-based nomogram for in-hospital mortality, utilizing eight easily accessible clinical features at admission, was successfully developed, which demonstrated a relatively good predictive performance, achieving a favorable ROC of 0.755 in internal validation. Further, DCA curves suggested great clinical applicability of the LR-based nomogram in both the training set and internal validation set. Several retrospective studies have utilized critical illness scoring systems (e.g., SOFA score) to predict the in-hospital mortality risk of patients with hip fractures, which were also included in the nomogram in the present study (28–30). CHA2DS2-VASc and HAS-BLED scores were also used to identify their predictive ability for the in-hospital mortality in the present study. However, in the training set, the nomogram outperformed APS III, SOFA score, CHA2DS2-VASc score, and HAS-BLED score when compared by the means of NRI, IDI, and the DeLong test (Table 2).

### The clinical perspectives about the selected features

This is the first study to focus on the in-hospital mortality prediction of the hip fracture population complicated by AF, in which we revealed eight features of the most importance associated with in-hospital mortality in this population, including SOFA score, age, undergoing hip fracture surgery, congestive heart failure (CHF), myocardial infarct (MI), aniongap_min, PT_min, and INR_max. Among these features, the MI history overwhelmed others in aspect of feature importance (Figure 3A). As for MI, it has been demonstrated as the complication most associated with 1-year mortality for patients with hip fracture, which is common and has a poor prognosis after hip fracture (31, 32). Actually, the most common perioperative complication associated with a hip fracture is myocardial injury, which is seen in at least 20% of patients at hospital presentation (33, 34). Ran et al. observed that the incidence of post operative acute myocardial infarct (AMI) in older adult hip fracture patients combined with coronary heart disease is 11.1%, which may be associated with anesthesia type, intraoperative bleeding, and intraoperative hypotension (35). In the present study, the history of the MI serves as a risk factor for the in-hospital mortality of hip fracture patients with AF. Perhaps, the perioperative AMI may partly contribute to such high mortality for patients with a poor coronary artery condition, especially for those who have suffered from MI previously and unstable perioperative vital signs. CHF is also known as a key risk factor for the in-hospital mortality among critical patients (36). In older adult patients with hip fractures, it was an independent factor of short and long-term mortality (37). Moreover, CHF greatly affects the patient’s cardiac function and predisposes them to cardiac accidents, which may result in reduced physical activity, longer postoperative recovery times, and higher rates of deterioration. All the above demonstrate that CHF is really a dangerous complication for critically ill patients with hip fractures.

Although proposed to primarily evaluate organ dysfunction, the SOFA score has been considered a significant predictor of in-hospital mortality in different clinical scenarios (38, 39), such as sepsis, myocardial infarction, and heart failure. For hip fracture or AF patients, the SOFA score also worked as a key indicator to predict the mortality risk, which further validates the present study (40, 41). In the present study, several biochemical indicators have been identified as being associated with the in-hospital mortality of these patients. Firstly, in this study, the INR was the second-highest risk factor after the SOFA score. The INR serves as a critical biomarker for assessing the coagulation status in patients. Perioperative thromboprophylaxis is now a routine practice in the management of older adult patients undergoing treatment for hip fractures (42). Meanwhile, regarding patients with combined AF, oral anticoagulants are used to keep INR levels between 2.0 and 3.0. Elevated INR has been demonstrated to be relevant with increased mortality in an ICU (43). Varady et al. observed that elevated INR was associated with an increased risk of reoperations, readmissions, and death (*p* < 0.001 for all) after hip fracture surgery, with the most pronounced effects observed at INRs >1.5 (44). PT is a common coagulation laboratory indicator, which holds the third rank of importance and displayed a positive effect in our study. In a previous study, it was deemed an indicator associated with prognosis among critically ill patients (45, 46). Moreover, a machine learning-based prediction model for long-term mortality in hip fracture patients enrolled PT as a risk factor (47). Prolonged PT may increase the risk of death in hip fracture patients with AF, which reflects the absence of normal coagulation ability related to a high bleeding risk.

Additionally, anion gap denotes the difference between the concentration of unmeasured anions and cations in plasma, which helps evaluate acid–base disorders. Previous studies have discovered a relationship between anion gap and mortality in clinically ill patients in an ICU. Wang et al. (48) found that in patients with cerebral infarction, an early post-rtPA increase in anion gap (>14 mmol/L within 48 h) predicted significantly elevated mortality rates over the short and long term (overall, 1-year, and 4-year). Besides, a large-scale cohort study suggested a positive association between postoperative anion gap levels and short- and long-term mortality among patients after cardiac surgery (49). In this study, high levels of anion gap remain used to identify hip fracture patients combined with AF at risk of hospital mortality in an ICU. Hip fracture Surgery is the only protective factor for the target population in our study. Existing evidence has shown that earlier surgery is linked to improved outcomes (50, 51). All the above support our study to regard these risk factors in the final model.

### The clinical prospect of the present study

This is the first nomogram-based prediction model for in-hospital mortality in hip fracture patients with AF, in which XGBoost ML model was utilized to assist in selecting features. The final model of the top 8 features screened by the XGBoost algorithm presents a favorable predictive performance and has the potential to guide clinical decision-making to obtain clinical benefit. To better meet clinical demands, we further developed an online application on https://fkd9fq-zhenli-li.shinyapps.io/1234/ (Supplementary Figure S3). However, our study also has several limitations. Firstly, the number of patients with hip fractures and AF in the MIMIC database is relatively small in the training set. Secondly, an AUC of 0.715 was observed in external validation, which is relatively lower than our expectation. The difference in predictive performance between internal and external validation may be partially attributed to the differences in basic patient information in MIMIC and eICU databases, which remains to be further analyzed. However, the DCA curve performed well in the range of 5 to 100% of the risk threshold to obtain the clinical benefit. The prediction model should be further trained and validated in a larger cohort of patients with both hip fractures and AF to increase generalizability. Thirdly, there is quite limited surgery information in the used databases. Thus, we cannot discuss the impact of surgical information on the prognosis of hip fractures, including surgery timing, and postoperative management may vary significantly across different cases, directly influencing fracture healing and patient recovery. Finally, potential bias may be observed due to the fact that the majority of patients were white. Collectively, further supplementation and validation are warranted to improve the predictive performance and generalizability of the ML model.

## Conclusion

In the present study, we developed and validated a predictive nomogram for in-hospital mortality in hip fracture patients complicated by AF in an ICU, employing the ML method to select the important features. It provided a robust and accessible method for evaluating the mortality risk, thereby facilitating effective clinical decision-making for such patients. Moreover, it was also validated by the eICU-CRD database and showed a relatively good performance. However, other external validation is still needed to further validate the model and explore its applicability across broader patient populations.

## Data Availability

The original contributions presented in the study are included in the article/Supplementary material, further inquiries can be directed to the corresponding author.
