# Supplementary material for: Development and validation of a nomogram for predicting in-hospital mortality in older adult hip fracture patients with atrial fibrillation: a retrospective study
Source: Front Med (Lausanne). 2025 Jul 23;12:1605437. doi: 10.3389/fmed.2025.1605437 (PMC12326479; doi:10.3389/fmed.2025.1605437)
Supplement: Supplementary file 1 [file Data_Sheet_1.zip › presentation/Supplementary Figures.pdf]

Title of the article	Development and validation of a nomogram for predicting in-hospital mortality in elderly hip fracture patients with atrial fibrillation: A retrospective study
Journal name	*Frontiers in Medicine*
Author names	Zhenli Li, Jing He, Tiezhu Yao, Guang Liu, Jing Liu, Ling Guo, Zhengkun Guan, Ruolian Gao, and Jingtao Ma
Affiliation of the corresponding author	The Fourth Hospital of Hebei Medical University
E-mail of the corresponding author	jingtm0502@163.com

Figures

Figure Legends

Figure S1. The specific usage of anticoagulant and antiplatelet drugs among the training set(A) and the usage of the anticoagulant and antiplatelet drugs between the MI and non-MI groups in the training set(B). MI, myocardial infarct; NOAC, new oral anticoagulants.

Figure S2. The ROC curves of the HAS-BLED scoring system and CHA2DS2-VASc scoring system in the training set(A).The ROC curves of the SOFA scoring system and APS III scoring system in the training set(B). ROC, receiver operating characteristic.

Figure S3. The external validation of the established nomogram based on the patients from eICU-CRD. The ROC curve(A), the calibration curve(B) and the decision curve analysis curve(C). ROC, receiver operating characteristic.

Figure S4. The web application of the dynamic nomogram in the study.

Figure S1

A

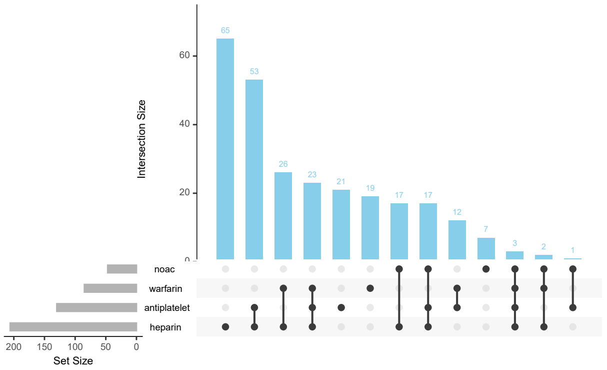

B

Patient demographics and baseline characteristics

Characteristic	Myocardial_infarct No N = 231	Myocardial_infarct Yes N = 77	p-value
**Warfarin, n (%)**			0.941[1]
0	167 (72.3%)	56 (72.7%)	
1	64 (27.7%)	21 (27.3%)	
**Antiplatelet, n (%)**			<0.001[1]
0	146 (63.2%)	32 (41.6%)	
1	85 (36.8%)	45 (58.4%)	
**Heparin, n (%)**			0.675[1]
0	75 (32.5%)	27 (35.1%)	
1	156 (67.5%)	50 (64.9%)	
**NOAC, n (%)**			0.234[1]
0	199 (86.1%)	62 (80.5%)	
1	32 (13.9%)	15 (19.5%)	

[1]Pearson's Chi-squared test

Figure S2

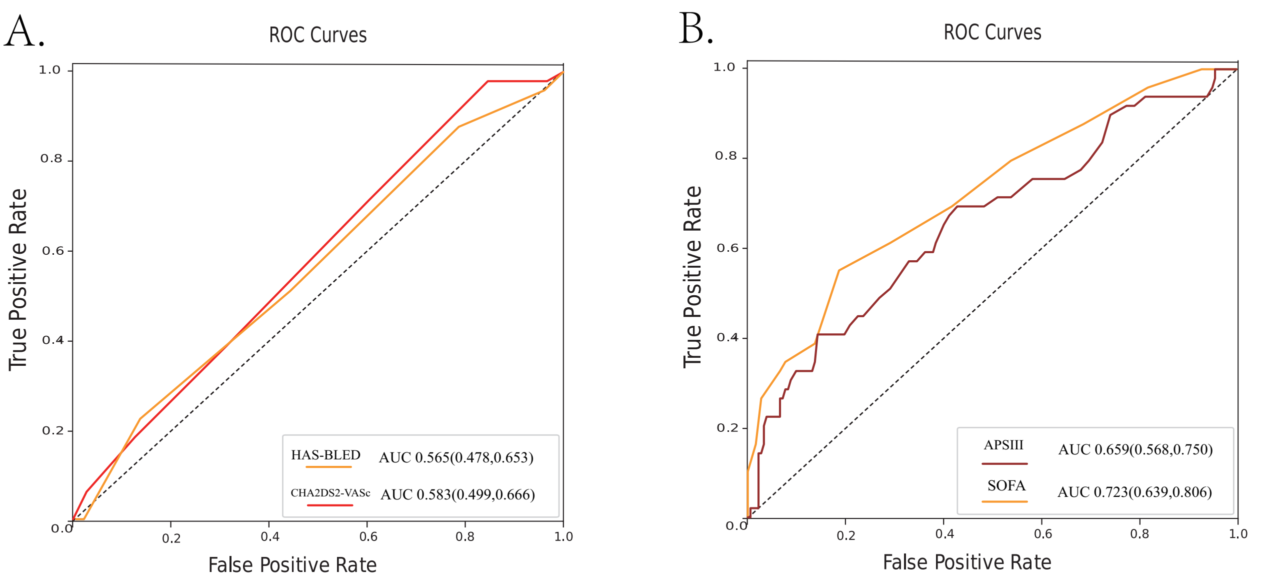
A.
ROC Curves
True Positive Rate
False Positive Rate
HAS-BLED
AUC 0.565(0.478,0.653)
CHA2DS2-VASc
AUC 0.583(0.499,0.666)
B.
ROC Curves
True Positive Rate
False Positive Rate
APSIII
AUC 0.659(0.568,0.750)
SOFA
AUC 0.723(0.639,0.806)

Figure S3

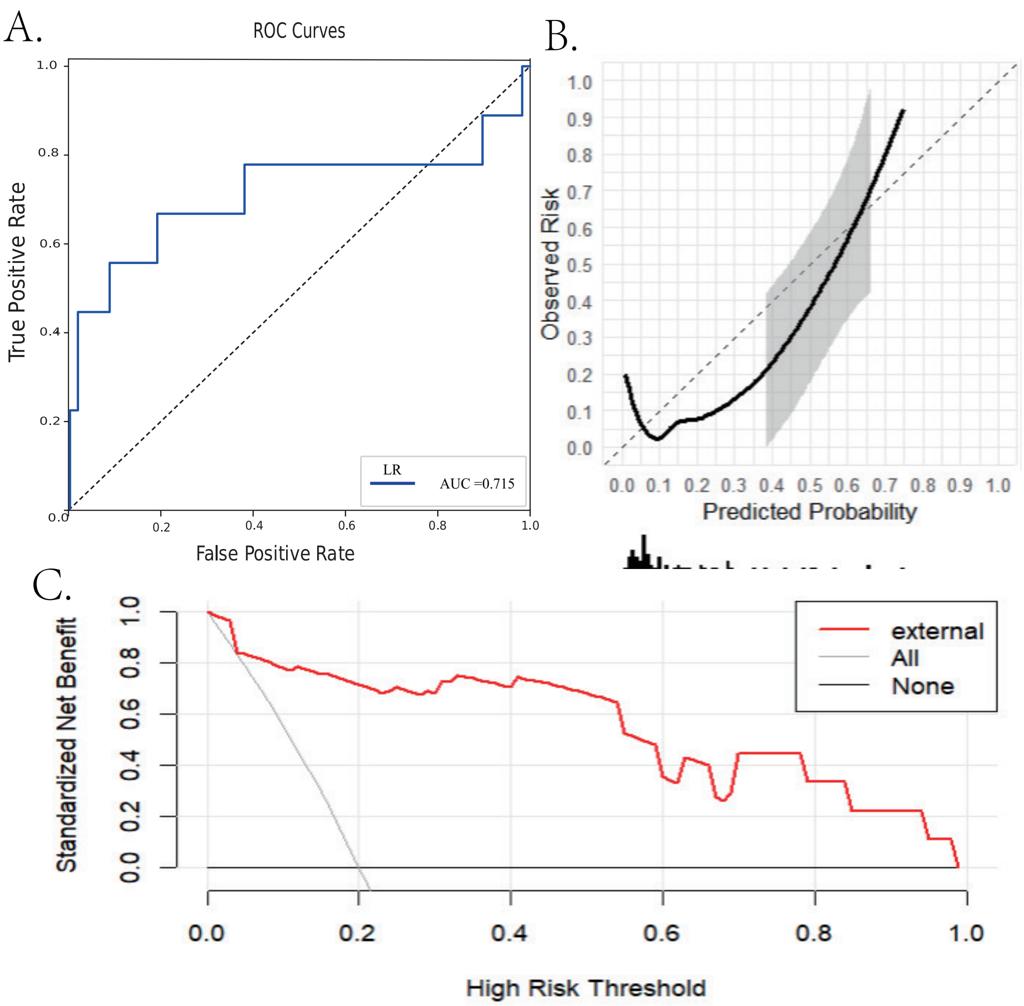
A.
ROC Curves
True Positive Rate
False Positive Rate
LR
AUC =0.715
B.
Observed Risk
Predicted Probability
C.
Standardized Net Benefit
High Risk Threshold
external
All
None

Figure S4

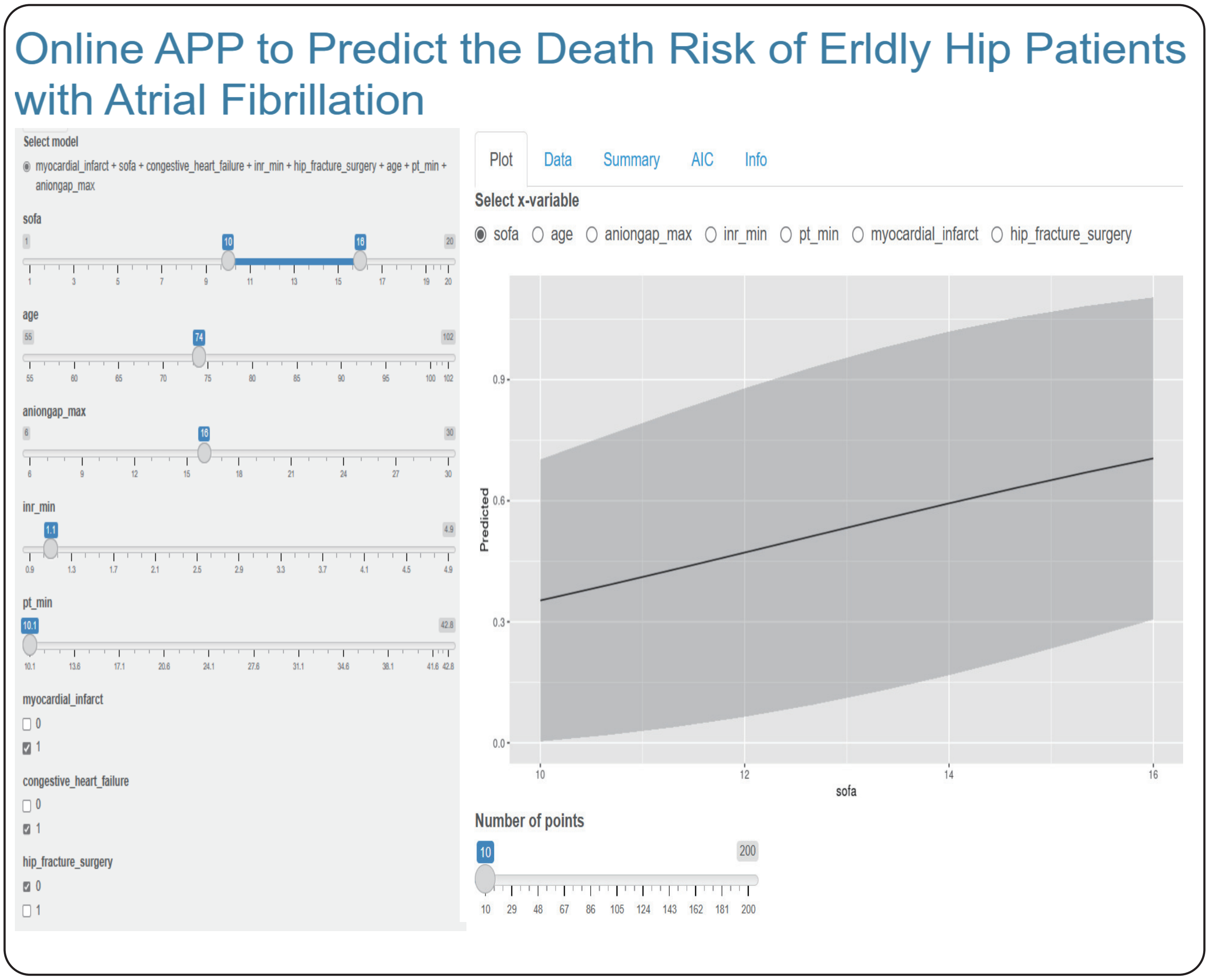
Online APP to Predict the Death Risk of Erldly Hip Patients with Atrial Fibrillation
Select model
myocardial_infarct + sofa + congestive_heart_failure + inr_min + hip_fracture_surgery + age + pt_min + aniongap_max
sofa
age
aniongap_max
inr_min
pt_min
myocardial_infarct
congestive_heart_failure
hip_fracture_surgery
Plot
Data
Summary
AIC
Info
Select x-variable
sofa
age
aniongap_max
inr_min
pt_min
myocardial_infarct
hip_fracture_surgery
Predicted
sofa
Number of points
